# Mitochondrial DNA copy number instability in ERBB2-amplified breast cancer tumors

**DOI:** 10.17179/excli2017-819

**Published:** 2018-01-22

**Authors:** Elmira Ebrahimi, Amir Almasi-Hashiani, Kimia Ghaffari, Reza Shirkoohi

**Affiliations:** 1 Cancer Biology Research Center, Cancer Institute, Imam Khomeini Hospital Complex, Tehran University of Medical Sciences, Tehran, Iran; 2 Department of Epidemiology and Reproductive Health, Reproductive Epidemiology Research Center, Royan Institute for Reproductive Biomedicine, ACECR, Tehran, Iran

**Keywords:** breast cancer, Epidermal Growth Factor Receptor, gene amplification, genomic instability, mitochondrial DNA

## Abstract

Increase in the copy number of *ERBB2*, a Tyrosine Kinase Receptor (TKR) leads to the overexpression of oncogene product and consequently uncontrolled cell proliferation which has been reported in different aggressive cancers with mitochondrial malfunctions. Although, amplification of *ERBB2* has been reported in different studies; however, the association between changes in mitochondrial DNA content and the *ERBB2* gene copy number is poorly understood. The relative mitochondrial DNA content of breast cancer tumor tissues of 70 patients who were referred to Imam Khomeini Hospital Complex was determined using quantitative Real-time PCR. Multiplex ligation-dependent probe amplification (MLPA) was conducted to evaluate the *ERBB2 *gene copy number variation and finally, two-sample Wilcoxon rank-sum (Mann-Whitney) test was used to investigate the possible association between mitochondrial DNA (mtDNA) content and the *ERBB2* gene amplification. Seventeen out of 70 breast cancer tumor tissues were found with *ERBB2* gene amplification. Comparison of the mitochondrial DNA content of the aforementioned samples with the rest of the cases showed a significant decrease in the mitochondrial DNA content of the *ERBB2*-amplified samples (P=0.01). Our data provided evidence that *ERBB2* have the potential to have a regulatory role over mitochondrial activity by controlling the mtDNA content.

## Introduction

Breast cancer is the most frequently diagnosed type of malignancy in women worldwide which accounts for about 29 % of all cancer types affecting females. According to the WHO estimation, there will be 246.660 new cases of breast cancer and 40.450 cases of deaths in the US in 2016 (Siegel et al., 2016[32]). The incidence rate of breast cancer is also rapidly increasing in Asia, Middle East and also in Iran (Harirchi et al., 2004[13]; Thyagarajan et al., 2013[36]). In the recent years, there has been a remarkable growth in the whole globe to improve the conventional cancer screening outcomes by using cancer-related genetic markers. Although alteration in genetic and epigenetic in genomic DNA encoded oncogenes and tumor suppressor genes have been in the focus of many researchers for many years, however, changes in mtDNA content has recently been a topic for debate to be used as a molecular biomarker for early detection (Yu, 2011[42]). 

Mitochondria are semiautonomous, cytoplasmic compartments of eukaryotic cells that have crucial roles in energy metabolism, generating free radicals, calcium homeostasis and programmed cell death. They have their own DNA called mtDNA which is a circular, double-stranded DNA with 16569bp length. It encodes 13 subunits of respiratory chain complexes, 2 ribosomal RNAs and a set of 22 transfer RNAs that are needed for synthesis of mitochondrial proteins. There are ten to thousands copies of mtDNA in each eukaryotic cell. Different tissues and cell types have different mtDNA content depending on its physiological circumstances and microenvironment condition (Wang et al., 2006[40]; Yu et al., 2007[43]; Shen et al., 2010[31]; Jiang et al., 2014[14]). It has been proposed that changes in mitochondrial DNA are in association to mitochondrial malfunctions suggesting that it may contribute to diseases including cancer (Bai et al., 2011[2]). Mitochondrial malfunctions can prompt the cells to escape from the normal apoptotic pathway and to initiate the neoplastic alteration (Wang et al., 2006[40]; Yu et al., 2007[43]). 

Changes in the mitochondrial DNA content trigger retrograde signaling pathway which is a crosstalk between mitochondria and the nucleus. In the normal cells, gene expression needs high energy which is provided by oxidative phosphorylation supplemented by mitochondria. Therefore, changes in the mitochondrial DNA content or function could possibly lead to changes in the ATP content which may result in an altered nuclear gene expression profile (Guha and Avadhani, 2013[11]; Picard et al., 2014[28]; Guantes et al., 2015[10]).

One of the most well-known genes in breast cancer that the extent of its expression is used as a prognostic value is *ERBB2 *(Harari and Yarden, 2000[12]). *ERBB2* as a member of Epidermal Growth Factor Receptor family encodes a transmembrane glycoprotein (HER2) with 185 KDa weight. It has a tyrosine kinase activity. It modulates several cellular functions by binding to other ligand bound EGF receptor which subsequently activates downstream signaling cascades including the MAP kinase, phosphatidylinositol-3-kinase (PI3-K) and calcium signaling pathways (Kauraniemi and Kallioniemi, 2006[16]). *ERBB2* amplification and/or overexpression has been reported in 15-30 % of human breast cancer malignancies and has been shown to contribute in an increased risk of recurrent disease and poor clinical outcome by promoting many cellular functions including DNA synthesis, cell proliferation, and cell survival.

The crosstalk between *ERBB2 *and mitochondria has been indicated by different studies. Suppressing apoptosis is known as a preliminary function of HER2 to enhance cell survival (Carpenter and Lo, 2013[4]). *ERBB2* has a regulatory role over the respiratory function of the mitochondria by changing the oxidative phosphorylation to the aerobic glycolysis for cancerous cells as an energy source. Moreover, it has been indicated that *ERBB2* induces mitochondrial malfunctions in cardiac myocyte through its translocation to mitochondria and regulating mitochondrial respiratory function (Ding et al., 2012[7]). 

Today, there is not enough evidence suggesting a link between changes in the *ERBB2* copy number and variability in the mitochondrial DNA content. Therefore, this study aimed to find out the possible association between the mtDNA content and the *ERBB2* gene amplification.

## Materials and Methods

Seventy frozen tumor tissues which were histologically confirmed as a breast cancer, as well as 8 non-cancerous breast tissues, were randomly retrieved from Iran National tumor bank, Cancer Institute, Imam Khomeini Hospital, Tehran, Iran. Clinico-pathological status of each tumor tissue including size, stage, grade, as well as the status of the receptors including estrogen (ER), progesterone (PR), human epidermal growth factor receptor-2 (HER2/neu) and tumor-suppressor protein p53 was also recorded for further analysis. An informed consent form was obtained from all individuals who were enrolled in this study. This study was approved by the ethics committee of Tehran University of Medical Sciences, Tehran, Iran. 

### Total DNA extraction

Total DNA from all breast tissue samples was extracted according to the manufacturer instruction using QIAamp DNA mini kit (Qiagen). The quality of the extracted DNA was checked by running all samples on 1.5 % agarose gel electrophoresis. 

### Determination of mtDNA content

Quantitation of the mitochondrial DNA copy number relative to the nuclear DNA was carried out by using real-time PCR. Firstly, primers for mtDNA and beta-globin, as a nuclear DNA, were designed using Primer3. Mitochondrial Primer sequences were selected from ND1 region which were as followed; mt-ND1-F- 5'-AACATACCCATGGCCAACCT-3', mt-ND1-R-5'-AGCGAAGGGTTGTAGTAGCCC-3' (Product size: 533-bp). The primer sequences of Beta-globin were: Betaglobin-F-5'-GAAGAGCCAAGGACAGGTAC-3'and Betaglobin-R-5'-CAACTTCATCCACGTTCACC-3' (Product size: 268-bp). Real-time PCR was then performed on Rotor gene Q (QIAGEN, USA). Each reaction was carried out in triplicate and in a total volume of 10 µl containing 2 µl nuclease free H2O, 1 μl cDNA (3.12 ng/µl), 5 μl of 2⨯ SYBER Premix Ex Taq II (TaKaRa, Japan), and 1 μl of forward and reverse primers (5 pmol/µl). The cycling reaction was initiated with 30 sec at 95 °C as a pre-denaturation step and then followed by 10 sec at 95 °C, 30 sec at 55 °C and 34 sec at 72 °C.

In each PCR run, 8 serially diluted DNA samples from 50 ng to 0.39 ng were generated as a standard curve. The following equation was then used to quantify the mtDNA copy number compared to beta-globin: 2 ^ΔCt ^(ΔCt = Ct β-globin-Ct ND1) (Kim et al., 2011[17]). 

### Multiplex Ligation-dependent Probe Amplification (MLPA)

*ERBB2* gene amplification status was determined using SALSA MLPA P078-C1 Breast Tumor probe kit (MRC, Holland). In each PCR reaction, three DNA samples from normal breast tissues were included. The no-template tube containing TE buffer (0.1 mM EDTA+10 mM Tris-HCl, pH 8.2) was also included in each PCR reaction. All the procedure including denaturation, hybridization, ligation, and PCR reactions were conducted in a Peqlab thermocycler (Germany). PCR products were subsequently separated on an ABI3130 capillary sequencer (Applied Biosystems, USA) (Ghaffari et al., 2016[9]).

### Multiplex Ligation-dependent Probe Amplification analysis

In order to analyse the *ERBB2* gene copy number variation status GeneMarker ver 1.6 (softgenetics, USA) was used. As there are 4 *ERBB2* probes included in this kit, the mean value was calculated. The mean value below 0.7 was considered as a lost, while values between 0.7-1.3 and >1.3 were assigned as normal and amplified, respectively (Ding et al., 2012[7]; Ghaffari et al., 2016[9]).

### Statistical analysis

A nonparametric test (Mann-Whitney test and Kruskal-Wallis test) was used to compare the mtDNA content in 2 case and control groups and the pathological findings. The same test was used to investigate the association of mtDNA content with the *ERBB2* amplification and or deletion. Also, likelihood-ratio chi2 was used to evaluate the association between *ERBB2* (17q) and HER2. Two-sided *P* less than 0.05 was considered as statistically significant. 

## Results

### Demographic data for clinico-pathological features of breast tumors

All subjects included in this study were females with an average age of 49.26 (+ 13.19). Concerning the clinico-pathological features of the tumors 22.85 %, 44.28 % and 31.42 % belonged to grade I, grade II and grade III, respectively. For the tumor stage, 2 (2.85 %) belonged to stage I, 41 (58.57 %) belonged to stage II, 26 (37.14 %) belonged to stage III. Concerning the expression of the hormone receptors 44 (62.85 %), 36 (51.42 %) and (21.42 %) were positive for ER, PR, and HER2, respectively. Tumor aggressiveness biomarker, p53, was positive for 26 (37.14 %) tumor samples (Table 1(Tab. 1)).

### Determination of the mtDNA Copy Number

**Figure 1:**
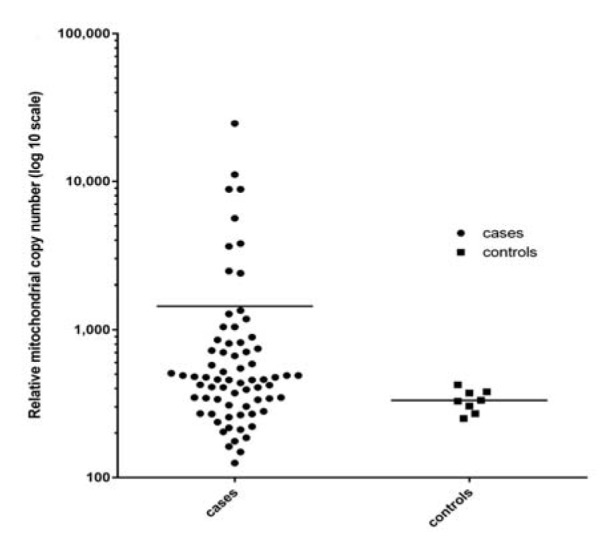
Scatter plot analysis illustrating the relative mtDNA content of breast tumor samples compared to normal breast tissues. The mean value of mtDNA content among cases was 1445 with max. mtDNA content of 24662 and min. of 125.4. The mean value in control group was 333 (max.: 424.6 and min.: 250.7).

In this study, distribution of the mtDNA copy number showed a wide spectrum compared to normal ones. After normalizing mitochondrial DNA content against the nuclear DNA content the average copy number of the mtDNA in the tumor tissue and the normal tissue group was 1445 ± 3473 and 333 ± 58.11, respectively. The difference between the mtDNA content of the tumor-tissue and the normal tissue group was statistically significant (*P* = 0.044) (Figure 1(Fig. 1)). 

Analyzing MLPA results we found 17 tumor samples with the *ERBB2* gene amplification which accounts for about 24 % of all samples. Studying the mtDNA content of the tumor samples according to the *ERBB2* amplification status, a significant association was found in the *ERBB2* amplified samples and a decreased level of the mtDNA content (*P*= 0.01). 

Interestingly, based on Immunohistochemistry (IHC) test results, 13 out of 17 samples were determined positive for HER2 protein expression (Table 2(Tab. 2)). 

Analyzing the possible factors affecting the mtDNA including patient age of onset, tumor size, stage, grade, as well as the status of the receptors including estrogen (ER), progesterone (PR), human epidermal growth factor receptor-2 (HER2/neu) and tumor-suppressor protein p53 no significant association was found (Table 3(Tab. 3)). 

The same result was found while we investigated the possible correlation between mitochondrial DNA content according to the ERBB2 status and the clinico-pathological features of the tumors (Table 4(Tab. 4)).

## Discussion

This project describes the outcome of the study on 70 breast cancer tumor tissues for investigating the possible role of *ERBB2* in regulating the mtDNA copy number variation. According to our observations, a significant decrease in the mtDNA content was seen in the *ERBB2* amplified samples which can prove the possible association between the *ERBB2* gene amplification and mitochondrial genetic content.

**Figure 2:**
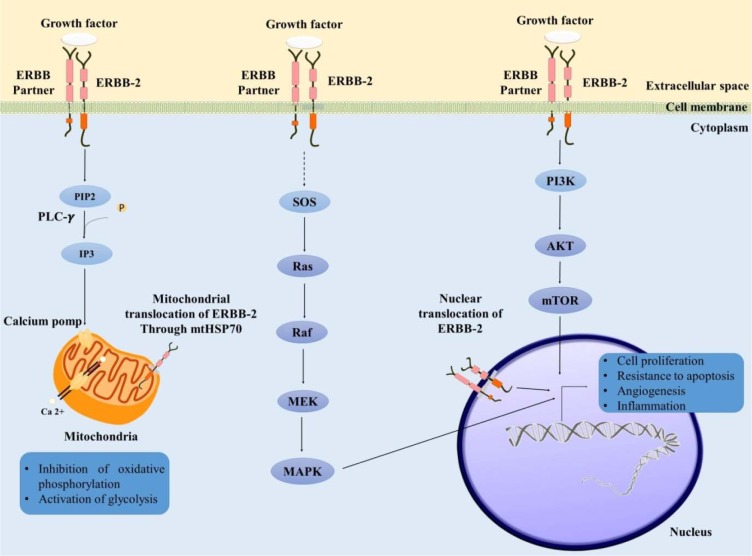
Schematic diagram of ERBB2 cellular signaling pathways known to regulate mitochondrial function. ERBB2 modulates several cellular functions by binding to other ligand bound EGF receptor which subsequently activates downstream signaling cascades including the MAP kinase, phosphatidylinositol-3-kinase (PI3-K) and calcium signaling pathways. It has been demonstrated that nuclear ERBB2 in the presence of unknown transcription factors activates the COX-2 gene. Translocation of ERBB2 to the mitochondria has also been observed in cancer cells and has been linked to decrease in ATP production and increase in cellular glycolysis.

Changes in the mitochondrial DNA, including alterations in mtDNA content, have been indicated throughout cancers and it has been denoted to contribute in mitochondrial malfunctions and disease (Malik and Czajka, 2013[23]). Herein, we found that the overall mtDNA content of the tumor tissues was significantly higher compared to normal ones (0.0436). While statistical analysis showed a significant increase in mitochondrial genetic content among tumor samples however a wide variation was seen in this group reinforcing instability in the mtDNA copy number preservation. Our findings were similar to the observations reported for other cancers types including brain (Liang and Hays, 1996[20]), head and neck (Jiang et al., 2005[15]), thyroid (Mambo et al., 2005[24]), lung (Lee et al., 2005[19]), and esophageal (Tan et al., 2006[34]). Although some studies have indicated a significant increase in mtDNA copy number in malignant tissues, however, there are few reports with opposite results. Yu and coworkers have shown a significant reduction in mtDNA content of 59 breast cancer tumor tissues compared to their adjacent normal ones (Yu et al., 2007[43]). Also, Fan and coworkers reported a significant decrease in 102 breast cancer tumor tissues compared to their corresponding normal tissues (Fan et al., 2009[8]). A similar phenomenon was also reported by another researcher in 2006 (Tseng et al., 2006[37]). The existence of such discrepancy shows that regulation of mitochondrial DNA copy number is a complex process controlled by different factors. Studies have demonstrated that different loci both on the nuclear and mitochondrial genome are responsible for maintenance of mitochondrial DNA content. Additionally, it has been demonstrated that the content of mtDNA is influenced by the activation of many signaling pathways (Rohlenova et al., 2016[29]). Constitutive activation of phosphoinositide 3-kinase/protein kinase B (PI3K/ AKT) through *ERBB2* amplification/overexpression has been shown to have effects on mitochondrial function (Rohlenova et al., 2016[29]). *ERBB2* also controls mitochondrial function by activating specific Her-2 related signaling pathways that subsequent to this process is cancer cell survival and proliferation (Rohlenova et al., 2016[29]; Victorino et al., 2016[38]). Subcellular translocation of ERBB2 to mitochondria (Ding et al., 2012[7]) and to the nucleus (Wang and Hung, 2009[39]) of cancer cells has recently been characterized by these researchers. These findings reinforce the role of ERBB2 in regulating mitochondrial function (Figure 2(Fig. 2)).

Moreover, frequent amplification of *ERBB2* has been reported in breast cancer tumors with high potential for cell proliferation, cell motility, invasiveness, and distant metastases. In addition, enhanced angiogenesis and abridged apoptosis in breast tumors have been shown to correlate with *ERBB2 *gene amplification (Owens et al., 2004[27]; Yaziji et al., 2004[41]; Kim et al., 2008[18]; Birnbaum et al., 2009[3]; Ross et al., 2009[30]; Meinhardt et al., 2015[26]; Chamizo et al., 2016[5]). Results of our observations proved the *ERBB2* amplification in breast tumors by the appliance of the MLPA technique. The rate of 24 % of *ERBB2* amplification in this study was similar to the range of previous reports (20-30 %) (Owens et al., 2004[27]; Yaziji et al., 2004[41]; Kim et al., 2008[18]; Birnbaum et al., 2009[3]; Ross et al., 2009[30]; Meinhardt et al., 2015[26]; Chamizo et al., 2016[5]). Interestingly, in our study, the mtDNA content in the *ERBB2 *amplified samples was significantly decreased (P=0.01). Reduction in the mtDNA copy number has been reported to correlate with the decreased mitochondrial activity. It has been demonstrated that reduction in the mtDNA copy number under hypoxic condition causes cells to shift from oxidative phosphorylation to glycolysis. A consequence of this process is a significant decrease in the Reactive Oxygen Species (ROS) production and leading cells to achieve immortalization which is known as the hallmark of cancer (Lin et al., 2008[21]). A drastic decline in the mtDNA content has also been reported to correlate with a reduction in the mitochondrial enzymes activity participating in energy metabolism (Meierhofer et al., 2004[25]). Moreover, about 76 % of our samples with *ERBB2* gene amplification were determined as HER2 positive using Immunohistochemistry (IHC) technique. Dickinson and coworkers in an experimental study demonstrated that “cancer cells failed to expand their mtDNA copy number and increase their respiratory capacity, which is underpinned by uncoordinated expression of the nuclear-encoded mtDNA replication factors” (Dickinson et al., 2013[6]). It has been shown that replication of mtDNA coincides with the up-regulated expression of DNA polymerase subunit gamma (POLG) replication factor (Thundathil et al., 2005[35]; Spikings et al., 2007[33]) which is indirectly controlled by the overexpression of ErbB-2/HER2 (Kim et al., 2011[17]; Lu et al., 2011[22]). 

In this study, the possible association of the mtDNA content of breast tumor tissues with the clinico-pathological features was also investigated; in regard to hormone receptors-ER and PR- even though we did not find a relationship between the two factors, however, the results of other studies were also contradictory. While Lin and co-workers have indicated higher mtDNA copy number in ER^+^/ PR^+^ tumors (Lin et al., 2008[21]), other studies (Tseng et al., 2006[37]; Yu et al., 2007[43]) have specified a higher mtDNA content in the ER-negative and PR-negative tumor samples. The presence of such controversy might be due to different studied subtypes of breast cancers or due to different sample size or other participating factors in this phenomenon that are not yet well characterized. Concerning the therapeutic biomarker p53 although a regulatory role in mitochondrial biogenesis has been reported (Achanta et al., 2005[1]) however, the association between p53 and changes in the mtDNA content did not result in this study. Concerning the stage of tumor progression, in a study, a significant decline in the mtDNA content of blood samples of patients with a breast cancer in stage I has been observed. They described that depletion of mtDNA in low stages could possibly prevent cancer cells from apoptosis (Fan et al., 2009[8]). Yet, in this study, mtDNA content did not change by the progression of the tumors in different stages. In regard to the disease age of onset similar to our findings, Fan et al. failed to find a link with mtDNA content. Knowing this, however, reduced mtDNA copy number has been reported in patients aged over 50 (Yu et al., 2007[43]). 

Overall, in this study amplification of *ERBB2* gene was correlated with decreased mtDNA content. Since the activation of *ERBB2* and downstream signaling pathways are involved in both mitochondrial bioenergetics machinery and programmed cell death, therefore our finding gives rise to this idea that *ERBB2* might mediate its action by regulating mitochondrial function through changes in mitochondrial genetic content. 

## Conflict of interest

All authors declare no conflict of interest.

## Figures and Tables

**Table 1:**
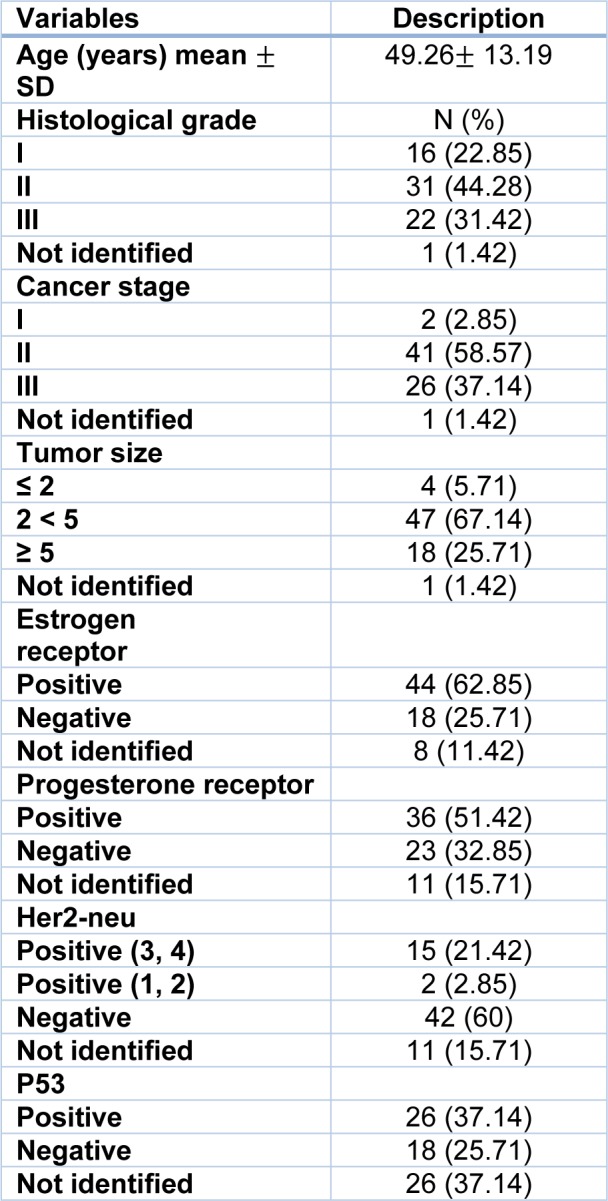
Demographic and disease condition data of 70 breast cancer patients included in this study

**Table 2:**
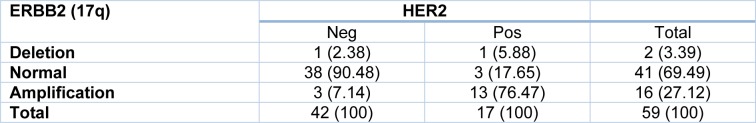
The association between ERBB2 (17q) and HER2 by likelihood-ratio chi2 (p<0.001)

**Table 3:**
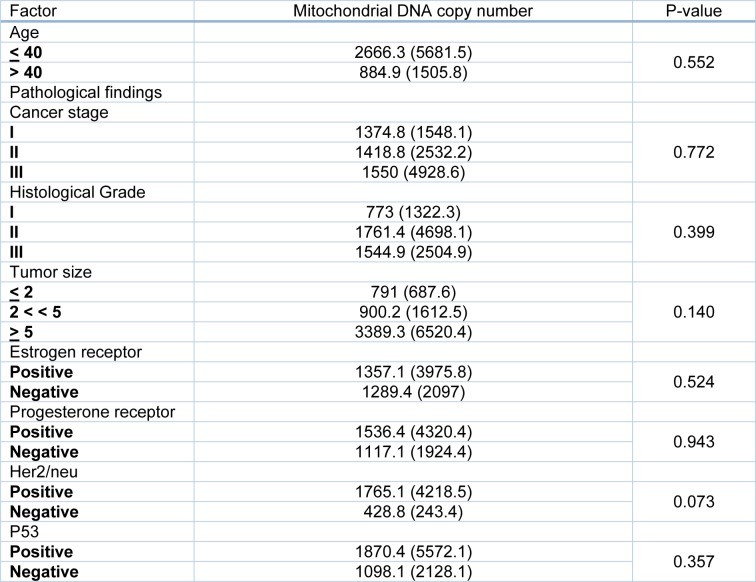
The possible factors affecting mitochondrial DNA content

**Table 4:**
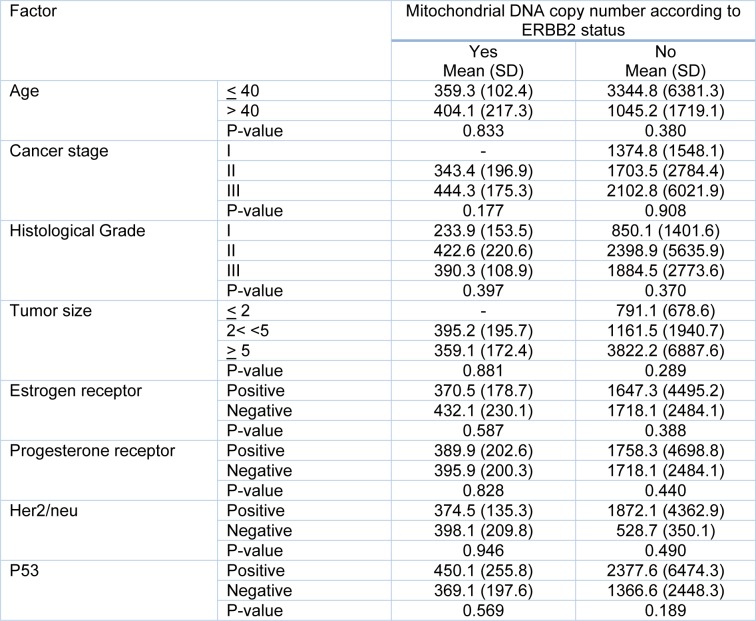
Possible factors affecting mitochondrial DNA content according to the ERBB2 status
